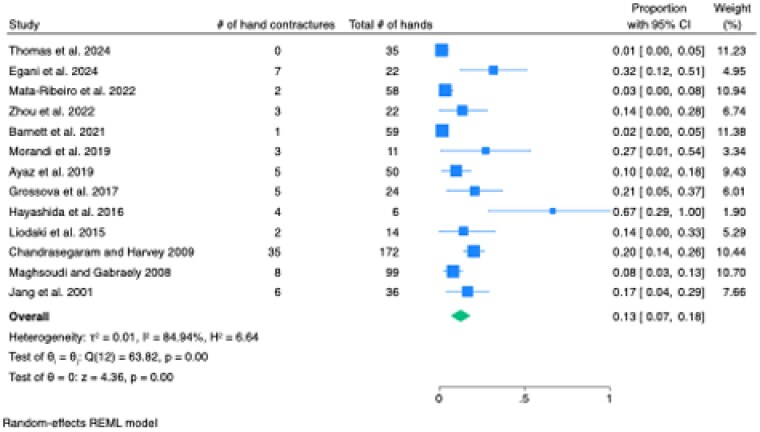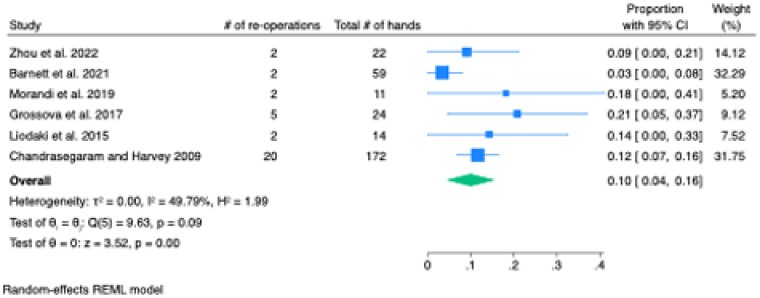# 532 Contractures Following Acute Hand Burn Surgery: A Systematic Review and Meta-analysis

**DOI:** 10.1093/jbcr/iraf019.161

**Published:** 2025-04-01

**Authors:** Hilary Liu, Ana Reis, José Arellano, Tiffany Jeong, Justine Kim, Teun Teunis, Guy Stofman, Francesco Egro

**Affiliations:** University of Pittsburgh Medical Center; University of Pittsburgh Medical Center; University of Pittsburgh Medical Center; University of Pittsburgh Medical Center; University of Pittsburgh Medical Center; University of Pittsburgh Medical Center; University of Pittsburgh Medical Center; University of Pittsburgh Medical Center

## Abstract

**Introduction:**

Hand burns are a common and debilitating injury that often require surgical intervention. Despite advancements in surgical techniques and postoperative care, post-burn scar contractures remain a prevalent complication, significantly impairing hand function and quality of life. The rate at which these contractures develop following acute hand burn injury varies widely across studies. This study aims to determine the rate of contracture following surgical treatment of acute hand burns.

**Methods:**

A systematic review and meta-analysis was conducted and reported according to PRISMA guidelines, and the protocol registered on PROSPERO. The results were limited to English-language articles from 2000-2024 with extractable data on the incidence of contracture occurrence after hand burn injuries that required surgical intervention.

**Results:**

Of the 2494 retrieved articles, 13 qualified for inclusion, reporting on 608 hand burns in 473 patients (72.4% male, 27.6% female). Most patients received burn excision and the application of a split-thickness or full-thickness skin graft (97.9%; n=595), with a small number receiving a free medial plantar flap (1.87%; n=12) or heterodigital flap (0.16%; n=1). The contracture rate was 12.6% [95% CI: 6.9, 18.3] (Figure 1). The rate of re-operation for the release of these contractures was 10.0% [95% CI: 4.4, 15.5] (Figure 2).

**Conclusions:**

The systematic review and meta-analysis revealed a 12.6% rate of post-burn scar contracture following surgical intervention for acute hand burns. These findings underscore the ongoing need for improved surgical techniques and postoperative care strategies to reduce the incidence of contractures and enhance patient outcomes.

**Applicability of Research to Practice:**

This study provides key data on post-burn contracture rates in hand burn patients, highlighting the need for improved surgical and postoperative care. The findings serve as a benchmark for future research and emphasize the importance of exploring new techniques to reduce contracture incidence. The systematic review methodology can guide similar studies in burn care.

**Funding for the Study:**

N/A